# Genomics Insight into *cfr*-Mediated Linezolid-Resistant LA-MRSA in Italian Pig Holdings

**DOI:** 10.3390/antibiotics12030530

**Published:** 2023-03-07

**Authors:** Manuela Iurescia, Elena Lavinia Diaconu, Patricia Alba, Fabiola Feltrin, Carmela Buccella, Roberta Onorati, Angelo Giacomi, Andrea Caprioli, Alessia Franco, Antonio Battisti, Virginia Carfora

**Affiliations:** National Reference Laboratory for Antimicrobial Resistance, Department of General Diagnostics, Istituto Zooprofilattico Sperimentale del Lazio e Della Toscana “M. Aleandri”, 00178 Rome, Italy

**Keywords:** *Staphylococcus aureus*, LA-MRSA, linezolid, *cfr*, WGS, long reads, short reads, bioinformatics analysis, CC1, CC398

## Abstract

The *cfr* genes encode for a 23S rRNA methyltransferase, conferring a multiresistance phenotype to phenicol, lincosamide, oxazolidinone, pleuromutilin, and streptogramin A antibiotics. These genes have been described in staphylococci, including methicillin-resistant *Staphylococcus aureus* (MRSA). In this study, we retrospectively performed an in-depth genomic characterisation of three *cfr*-positive, multidrug-resistant (MDR) livestock-associated (LA) MRSA clonal complexes (CCs) 1 and 398 detected in different Italian pig holdings (2008–2011) during population studies on Italian livestock (2008–2014). We used a combined Illumina and Oxford Nanopore Technologies (ONT) whole genome sequencing (WGS) approach on two isolates (the 2008 CC1 and the 2010 CC398 isolates, but not the 2011 CC1 isolate). Interestingly, the three isolates presented different *cfr* variants, with only one displaying a linezolid-resistant phenotype. In isolate 2008 CC1, the *cfr* gene was identified within a Tn558 composite transposon-like structure flanked by IS elements located on a novel 44,826 bp plasmid. This represents the first report of CC1 LA-MRSA harbouring the *cfr* gene in its functional variant. Differently, *cfr* was chromosomally located in isolate 2010 CC398. Our findings have significant public health implications, confirm the need for the continuous genomic surveillance of *cfr*-positive zoonotic LA-MRSA, and backdate *cfr* presence in LA-MRSA from Italian pigs to at least 2008.

## 1. Introduction

Linezolid was the first oxazolidinone antimicrobial available exclusively for human use from April 2000, and it represents one of the few therapeutic options to treat methicillin-resistant *Staphylococcus aureus* (MRSA) skin and soft-tissue infections, osteomyelitis, and pneumonia [1]. Seven mobilisable oxazolidinone resistance genes, including *cfr*, *cfr*(B), *cfr*(C), *cfr*(D), *cfr*(E), *optrA*, and *poxtA*, have been described so far [2]. In particular, after being identified for the first time about 20 years ago in *Staphylococcus sciuri* of bovine origin in Germany [3], *cfr* genes have been found in 19 staphylococcal species from both human and animal sources as well as in other bacterial species [2]. These genes encode for a 23S rRNA methyltransferase, which confers a multiresistance phenotype (phenotype PhLOPS_A_), including resistance to phenicol, lincosamide, oxazolidinone, pleuromutilin, and streptogramin A antibiotic classes [4]. The *cfr* genes have been detected in both methicillin-susceptible *S. aureus* (MSSA) and MRSA isolates from human clinical samples worldwide [2], including Italy [5]. The *cfr*(B) gene has recently been described for the first time in MRSA isolates from French clinical human samples [6]. In livestock, *cfr* was first described in *S. aureus* of porcine origin in Germany in 2006 [7]. Since then, it has sporadically been detected in pig samples from Belgium [8], Spain [9], Portugal [10], the Netherlands [11], China [12], and South Korea [13], and in bovine samples from Egypt [14]. These genes are most frequently located on mobile genetic elements (MGEs), especially plasmids, which were proven to harbour *cfr*-carrying transposons and translocatable units (TUs), playing a crucial role in their dissemination [15]. Additionally, TUs and integrative and conjugative elements (ICEs) have also been detected at a chromosomal level [2].

In this study, we retrospectively report the in-depth genomic characterisation of three *cfr*-positive, multidrug-resistant (MDR) livestock-associated (LA) MRSA isolates belonging to clonal complexes (CCs) 1 (two isolates) and 398 (one isolate), isolated between 2008 and 2011 from Italian pig holdings, combining Illumina short-read and Oxford Nanopore Technologies (ONT) long-read sequencing approaches.

## 2. Results

### 2.1. Antimicrobial Susceptibility Testing (AST)

The three LA-MRSA isolates were phenotypically MDR, displaying a common resistance pattern to beta-lactams, amphenicols, fluoroquinolones, and tetracyclines (Table 1). Both CC1 isolates (IDs 21554/1 and 55864/23) were also macrolide (erythromycin)- and lincosamide (clindamycin)-resistant. IDs 76669/3 (CC398) and 21554/1 (CC1) were pleuromutilin (tiamulin)-resistant. Based on the epidemiological cut-off value and clinical breakpoint of linezolid (MIC value > 4 mg/L), only one CC1 isolate (ID 21554/1) was phenotypically resistant to linezolid (MIC value = 8 mg/L); the other two were susceptible (MIC value = 1 mg/L).

### 2.2. WGS and Bioinformatics Analysis (Illumina Short Reads)

The previous results of multilocus sequence typing (MLST), *spa* typing, and staphylococcal cassette chromosome *mec* (SCC*mec*) typing were confirmed by the results obtained by in silico typing for all the three de novo assembled genomes. Isolates IDs 21554/1 and 55864/23 belonged to *spa* type t127, sequence type (ST) 1, CC1, and isolate ID 76669/3 belonged to *spa* type t034, ST398.

Except for linezolid resistance, all MDR phenotypes were consistent with the genetic analysis. All three isolates tested *cfr*-positive, but only one CC1 isolate (ID 21554/1) was phenotypically linezolid-resistant (Table 1).

**Figure 1:**
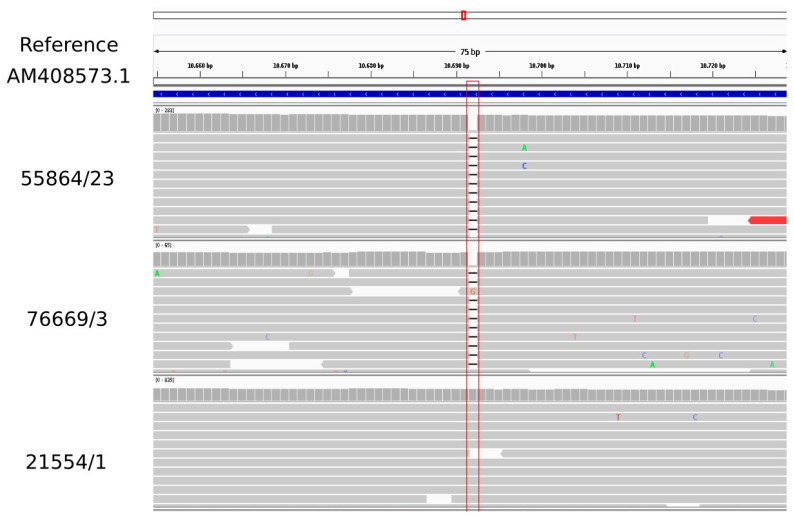
Visualisation of the variant calling analysis obtained for *cfr* gene of the three LA-MRSA isolates using as reference the Tn558 transposon of plasmid pSCFS6 (*S. warneri* (AM408573) (CGE database)).

The variant calling analysis indicated that both linezolid-susceptible isolates, ID 55864/23 (CC1) and 76669/3 (CC398), presented a *cfr* gene with a single nucleotide deletion corresponding with position 10,692 bp in the Tn558 transposon of plasmid pSCFS6. *S. warneri* (AM408573) was used as a reference sequence. This single base-pair deletion caused a reading frameshift and, consequently, a truncated protein. The linezolid-resistant CC1 LA-MRSA isolate (ID 21554/1) harboured a *cfr* sequence identical to AM408573 from *S. warneri* (Figure 1).

### 2.3. WGS and Bioinformatics Analysis (Hybrid Assembly)

The complete genomes of isolates ID 76669/3 (CC398) and 21554/1 (CC1) subjected to a hybrid (Illumina-ONT) assembly were obtained and analysed. The results of the Bakta annotation and MobileElementFinder analyses are reported in detail in Tables S1 and S2. In particular, the annotation of the assembly of isolate ID 76669/3 (CC398) identified 2649 coding sequences (CDSs), and *cfr* was located on contig 9 (160,836 bp; 30227..31275 nt) together with the *fex*A gene. These AMR genes were surrounded by IS elements within a Tn*558* composite transposon-like structure, including truncated *tnp*A (Δ*tnp*A), *tnp*-IS21, and *ist*B (IS21-558 element). A BLAST analysis revealed that contig 9 of isolate ID 76669/3 had the BLAST best match (according to Max Score) with the chromosome sequence of LA-MRSA ST398 (strain 17Gst354; CP073065.1) isolated from the nasal swab of a healthy Swiss farmer in 2017, showing a 96.00% coverage and a 99.78% identity. However, this strain did not harbour the *cfr* gene. The BLAST best match (98.00% coverage and 99.72% identity) with *cfr*-positive MRSA was with the chromosome sequence of the LA-MRSA ST398 strain (RIVM_M047065; CP096539) isolated from a human nasal swab in the Netherlands in 2019 [11].

The results of the average nucleotide identity (ANI) for the alignment coverage and identity of the complete sequence of isolate ID 76669/3 (2,843,032 bp) compared with the complete chromosomes of the LA-MRSA ST398 strain 17Gst354 (2,783,931 bp) and LA-MRSA ST398 strain RIVM_M047065 (2,806,671 bp) showed that they shared an identity of 99.76% and 99.86% with a 93.24% and 96.00% coverage, respectively.

**Figure 2:**
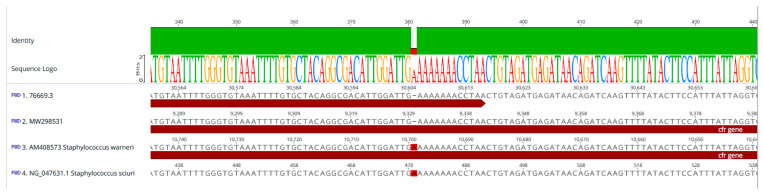
Multiple alignment of the contig-harbouring *cfr* of the CC398 isolate (ID 76669/3) (**1**) with (**2**) a 18,240 bp chromosomal region containing *cfr* (*cfr* gene cluster) from *S. aureus* G322 (MW29853), (**3**) a 22,010 bp reference sequence of the Tn558 variant of pSCFS6 plasmid from *S. warneri* (AM408573), and (**4**) a 1250 bp *cfr* sequence from pSCFS1 plasmid of *S. sciuri* (NG_047631).

From the NCBI database interrogation, we also observed that the same single base-pair deletion (c.381delA) found in the *cfr* gene of the CC398 LA-MRSA isolate ID 76669/3 was recently described in a *cfr* gene located in a 18,240 bp chromosomal region (*cfr* gene cluster; MW298531) of a CC398 LA-MRSA sample isolated in 2019 from a single pig nasal swab in Central Italy [18]. Multiple alignment confirmed this deletion by comparing our sequence with the *cfr* reference sequences of *S. sciuri* (NG_047631) and *S. warneri* (AM408573) (Figure 2).

An almost (99.99%) identical *cfr* gene cluster of MRSA G322 (MW298531), described by Fioriti and colleagues in 2021 [18], was identified within the transposon harboured on contig 9 of our isolate ID 76669/3 (21276..39515 nt). Both *cfr* gene clusters differed by only one nucleotide in the *fex*A gene encoding a phenicol resistance. In our isolate, this gene was identical to the *fex*A1 reference sequence in the ResFinder database (AJ549214) whereas the *fex*A gene within the *cfr* gene cluster of isolate MRSA G322 presented a single-point mutation (G to C) at position 1192 (G1192C). MRSA G322 remained phenotypically resistant to chloramphenicol and florfenicol [18].

**Figure 3:**
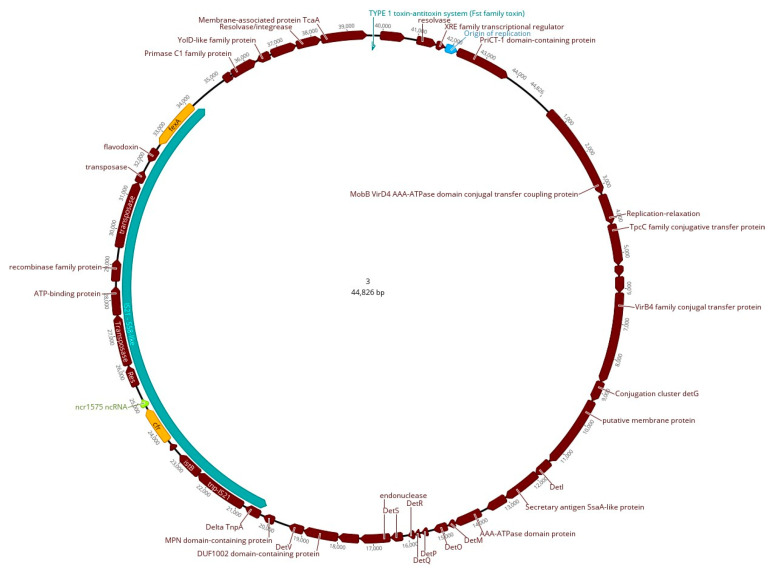
Graphical representation of the *cfr*-carrying plasmid (pMOL21554) of isolate ID 21554/1. Coding regions are indicated in dark red, *fex*A and *cfr* genes are marked in yellow, and the 1575 non-coding RNA (ncRNA) sequence is in light green. The Tn558 composite transposon-like structure (~13,774 bp) is indicated in greenish-blue, and the origin of replication is in light blue.

Resolved plasmids were obtained only for isolate ID 21554/1 (CC1) (circular contigs 3, 4, 5, and 6; Tables S1 and S2). The *cfr* gene was located on a novel 44,826 bp plasmid (contig 3; 23694..24743 nt) named pMOL21554 (Figure 3). Similar to contig 9 of isolate ID 76669/3, *cfr* was flanked by IS elements in this plasmid within a Tn*558* composite transposon-like structure (~13,774 bp). This structure presented an identity of 99.70% with a coverage of 100% with the reference sequence of IS21-558 (AM086211), composed by truncated *tnp*A (ΔtnpA), *tnp*-IS21, *ist*B (IS21-558 element), *cfr*, and *fex*A. The major difference with the reference sequence was an insertion in our IS21-558-like structure, which included additional mobile elements (25432..30683 nt) represented by two transposases, an ATP-binding protein, and two recombinases (Figure 3).

As reported for contig 9 of isolate ID 76669/3, this plasmid only harboured *fex*A and *cfr* as AMR resistance genes. Isolate ID 21554/1 harboured other AMR genes such as *bla*Z, *ble*O, *aad*D located in a rep20 plasmid and the *tet*(K) gene in a rep7a plasmid.

**Figure 4:**
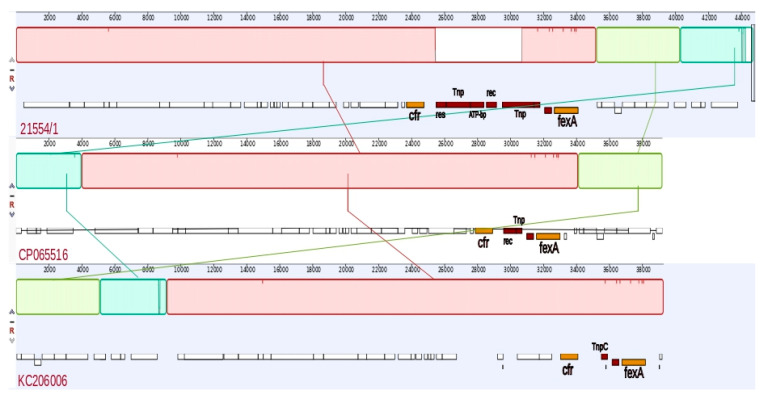
Graphical representation of the alignment of the *cfr*-carrying plasmid (pMOL21554) of isolate ID 21554/1 compared with selected already-deposited plasmid sequences publicly available: plasmid pY96A (CP065516.1) from an ST1 MSSA strain (pig; 2019; China) and plasmid pSA737 (KC206006) from an ST239 MSSA strain (human; 2017; Unites States).

The BLAST analysis revealed that pMOL21554 of isolate ID 21554/1 had the BLAST best match with: (i) a 39,287 bp plasmid (88% coverage and 100% identity) named pSA737 (KC206006) from ST239 MSSA (strain 004-737X) isolated in 2007 from a human clinical sample in the United States [19]; and (ii) a 39,212 bp plasmid (88% coverage and 99.99% identity) named pY96A (CP065516.1) from ST1 MSSA (strain GDY8P96A) isolated in 2019 from a pig nasal swab in China. The main differences between the 3 plasmid sequences were related to the presence in our plasmid of a 5249 bp (25430..30679 nt) region harbouring several transposable elements represented by a transposase, a recombinase, and an ATP-binding protein encoding gene. This region was absent in the two previously deposited plasmid sequences (Figure 4).

The complete sequences of the *cfr*-positive isolates and the resolved *cfr*-carrying plasmid were deposited in the European Nucleotide Archive (ENA) at EMBL-EBI under project accession number PRJEB59270.

## 3. Discussion

In this study, we report the in-depth molecular characterisation of CC1 (two isolates) and CC398 (one isolate) *cfr*-positive MDR LA-MRSA isolates of porcine origin detected for the first time in Italian animal production in 2008–2011 [16,17] by using the combined approach of short- and long-read sequencing. Based on previous population studies (2008–2014) conducted on Italian animal production and according to other European-wide studies on *cfr*-positive MRSA isolated from pig holdings, the occurrence of *cfr*-positive LA-MSSA and -MRSA in animal production is emerging, but seems to be sporadic [8,9,10,11]. Accordingly, in a recent study (2019–2020) conducted on dairy cattle farms located in Italian regions representing around 80% of Italian milk production, 22 LA-MRSA samples isolated from 316 bulk tank milk samples and belonging to different CCs tested negative for the presence of *cfr* [20].

The isolates characterised in this study presented different *cfr* variants/locations and linezolid-resistant phenotypes. The *cfr* gene was demonstrated to be located on a novel 44,826 bp plasmid in the CC1 isolate (ID 21554/1) and, to the best of our knowledge, this represents the first report of CC1 LA-MRSA harbouring the *cfr* gene in its functional variant; i.e., displaying both a microbiological and a clinical resistance. The presence of the *cfr* gene was previously detected only in a 39,212 bp plasmid (CP065516.1) from one CC1 MSSA sample isolated in 2019 from a pig nasal swab in China. Differently, in our CC398 LA-MRSA isolate (ID 76669/3), *cfr* seemed to be chromosomally located. This result was supported by a comparison with a previously deposited complete chromosome sequence of *cfr*-positive CC398 MRSA of human origin (strain RIVM_M047065; CP096539), considering the obtained coverage (96.00%) and identity (99.86%). This isolate, together with the CC1 isolate ID 55864/23, presented a frameshift mutation caused by a single base-pair deletion in the *cfr* gene and, consequently, a truncated protein, corroborating their phenotypic susceptibility to linezolid (MIC value = 1 mg/L). Prior to our findings, linezolid-susceptible MSSA and MRSA associated with *cfr* variants with a single base-pair deletion or mutation were isolated from two human clinical cases (one in Brazil [21] and one in the Netherlands [11]), and also from two nasal swabs of porcine origin (one in Italy [18] and the other in Australia (CP029172)). All of them belonged to CC398.

As for the genetic environment of *cfr* insertion sequences and mobile elements found close to *cfr*, those of the IS21 family (e.g., IS*21-558*) in particular have been reported to have an important role in its mobilisation [15]. The importance of the IS21 family appears to be consistent, irrespective of whether the *cfr* gene is found in plasmids or in chromosomal structures. IS*21-558*, also called ISS*au9*, was first identified on plasmid pSCFS3 from *S. aureus* of porcine origin in Germany [7], and a *cfr*-harbouring segment bound by two directly oriented copies of IS*21-558* was first identified in plasmid pSCFS6 from both *S. warneri* and *S. simulans* of porcine origin isolated in Denmark [22], suggesting that this structure could be transferred and spread among different staphylococcal species. In this regard, we identified in both isolates subjected to the hybrid assembly approach (IDs 76669/3-CC398 and 21554/1-CC1) a IS*21*-*558*-like structure near to the *fex*A and *cfr* genes. In plasmid pMOL21554 from isolate ID 21554/1, this structure was very similar (99.7% identity with 100% coverage) to the reference sequence of IS*21*-*558* (AM086211), suggesting that the *cfr* gene may have been transmitted through the Tn558 transposition. Considering the potential transmissibility of *cfr* and its emergence in animal production in Europe, these results have significant public health implications and backdate the *cfr* presence in LA-MRSA from Italian pigs at least to 2008. However, we could speculate that LA-MRSA of swine origin has had, so far, a mild attitude to acquire and maintain such resistance genes along the production cycles. This may reflect the low frequency of detection of *cfr*-positive MRSA observed so far in humans [11], even in case they may represent an important reservoir of exposure for animal production.

Moreover, it has to be considered that even if oxazolidinones and streptogramin A antibiotic classes are not used in food-producing animals, the selective pressure exerted by the non-prudent use of PhLOPSA antibiotic classes in both human and veterinary sectors (e.g., in humans, lincosamides and oxazolidinones; in veterinary medicine, phenicols, lincosamides, and pleuromutilins) may lead to the further dissemination of the *cfr* gene.

Although our identified plasmid harboured only *fex*A and *cfr*, the potential coexistence of *cfr* and other acquired AMR genes in the same plasmids is worrying as co-selection mechanisms and the persistence of the *cfr* gene may occur under selective pressure induced by the use of non-PhLOPSA antibiotic classes [15].

Notably, after the first European survey conducted in 2008 [23], a new European Union-wide baseline survey is to be carried out in the next years in order to provide updated and representative information on the hazard of LA-MRSA from pigs [24]. This type of harmonised study will help provide data at both European and national levels on linezolid-resistant LA-MRSA.

## 4. Conclusions

In conclusion, our findings confirm the need for the continuous genomic surveillance of *cfr*-positive zoonotic LA-MRSA and other oxazolidinone-resistant bacteria in livestock. This approach will help the understanding of the genetic environment and the transmission patterns of MGEs involved in the dissemination of powerful genetic determinants, mediating multiresistance to last-resort drugs for human medicine.

## 5. Materials and Methods

### 5.1. LA-MRSA Isolates

Three *cfr*-positive LA-MRSA samples (isolate IDs 21554/1, 76669/3, and 55864/23) were retrospectively characterised in depth by a hybrid WGS approach and a bioinformatics analysis. They had been isolated from two dust samples (IDs 21554/1 and 55864/23) and one nasal swab (ID 76669/3) collected from not epidemiologically related breeding, farrow-to-finish, and fattening (finishing) Italian commercial pig holdings (range: 2500–6500 heads) in 2008, 2011, and 2010, respectively. The three isolates, stored at the National Reference Laboratory for Antimicrobial Resistance (NRL-AR), Istituto Zooprofilattico Sperimentale del Lazio e della Toscana “M. Aleandri”, Rome (Italy), had been previously characterised by a DNA microarray analysis and molecular typing through *spa* typing, multilocus sequence typing, and SCC*mec* typing, as previously described [16,17].

These isolates were the only *cfr*-positive ones from a collection of LA-MRSA isolates (*n* = 433) available and screened at the NRL-AR, and were obtained from different animal production methods such as fattening pigs (*n* = 172), dairy cows (*n* = 118), veal calves (*n* = 142), and broiler chicken (*n* = 1) in the context of national population studies conducted in Italy between 2008 and 2014 [16,17,25,26,27,28,29].

### 5.2. Antimicrobial Susceptibility Testing (AST)

The three *cfr*-positive LA-MRSA isolates were previously tested for their antimicrobial susceptibility by broth microdilution (Trek Diagnostic Systems, Westlake, OH, USA). The MICs were interpreted according to the European Committee on Antimicrobial Susceptibility Testing (EUCAST; http://www.eucast.org (accessed on 20 June 2022) using epidemiological cut-offs. The following panel of drugs was tested: penicillin, cefoxitin, ciprofloxacin, chloramphenicol, clindamycin, erythromycin, gentamicin, kanamycin, streptomycin, linezolid, quinopristin/dalfopristin, fusidic acid, mupirocin, rifampicin, tetracycline, tiamulin, sulfamethoxazole, trimethoprim, and vancomycin [16].

### 5.3. Library Preparation and Whole Genome Sequencing (WGS)

DNA extraction and library preparation were performed as previously reported [30]. Briefly, genomic DNA was extracted using a QIAamp DNA Mini Kit (Qiagen, Hilden, Germany) following the manufacturer’s protocol. The libraries for short-read pair-end sequencing were prepared for the three isolates using a NexteraXT DNA library preparation kit (Illumina, Inc., San Diego, CA, USA) and sequenced on an Illumina platform (MiSeq sequencer, Illumina, Inc., San Diego, CA, USA)). In parallel, the libraries of two isolates (IDs 21554/1 and 76669/3) were prepared with a ligation sequencing kit (SQK-LSK109) and sequenced using a nanopore-based MinION device (ONT) [31].

### 5.4. Bioinformatics Analysis (Illumina Short Reads)

Illumina raw reads were analysed using an internal pipeline for the assembly, which included the following tools: FastQC v0.11.9, Trimmomatic v0.39 [32], Spades v3.13.0 [33], and Quast v5.0.2 [34]. In silico molecular characterisation was performed on all the assembled genomes for the MLST analysis [35,36] to confirm the ST. Center for Genomic Epidemiology (CGE) online tools with default parameters (http://www.genomicepidemiology.org/services/ (accessed on 19 July 2022)) were used to confirm the SCC*mec* type [37,38], the *spa* type [39], and the genetic basis of AMR [40,41,42]. The presence of *cfr* mutations was investigated by mapping the trimmed reads to the *cfr* reference sequence of the CGE ResFinder database (updated on 24 May 2022) AM408573 (*S. warneri*), and variant calling was performed using BWA mem v0.7.12 [43] and SAMtools v1.7 [44], and then visualised by IGV 2.0.1 [45].

### 5.5. Bioinformatics Analysis (Hybrid Assembly)

For the two isolates subjected to both short- and long-read sequencing, high accuracy base-calling was performed on the long reads obtained from the MinION device according to the ONT workflow. A hybrid (Illumina-ONT) assembly was implemented using the Unicycler pipeline [46] with default parameters [47]. The obtained assemblies were also annotated using the online version of the Bakta tool (https://bakta.computational.bio/ (accessed on 19 July 2022) [48]. Additionally, a manual curation for the obtained annotation was performed. The identification of the mobile genetic elements and their relation to the AMR genes and virulence factors was performed with the online version of the CGE tool, MobileElementFinder v1.0.3 (last accessed 19 July 2022) [49] using default parameters. From the hybrid assemblies, the obtained contigs containing *cfr* were also compared with the BLAST algorithm using the online tool against the nr/nt database with default parameters. The complete sequence of isolate ID 76669/3 was also compared with selected complete chromosome sequences of *cfr*-positive *S. aureus* obtained from publicly available databases showing the BLAST best match and calculating the ANI for the alignment coverage and identity using the BLAST algorithm [50].

The presence of mutations of the *cfr* genes was confirmed, and their genetic environment was investigated by comparing the multiple alignment of the contig-harbouring *cfr* of isolate ID 76669/3 with publicly available selected sequences harbouring *cfr* from: (i) *S. warneri* (AM408573) [22]; (ii) *S. sciuri* (NG_047631) [51]; and (iii) *S. aureus* G322 (MW29853) [18]. This was achieved using Geneious Prime 2023.0.1 software.

The graphical representation of the general structures and of the genetic regions of the *cfr*-carrying plasmid of isolate ID 21554/1 as well as the comparison with selected already-deposited plasmid sequences were performed using Geneious Prime 2023.0.1 and the Mauve algorithm for the alignment [52].

## Figures and Tables

**Table 1:** Genomic and phenotypic characteristics of the three *cfr*-positive LA-MRSA isolates analysed by WGS (Illumina short reads).

MRSA ID	Year of Isolation	Matrix of Origin	Host Production	*Spa* Type	ST	CC	SCC*mec* Type	AMR Phenotype	AMR Genotype
21554/1 ^§^	2008	Dust sample	Pig breeding	t127	1	1	V	CHL, CIP, TET, FOX, CLI, ERY, LIZ, PEN, TML, SYN	*ant*(9)-Ia °, *aad*D, *ble*O, *mec*A, *bla*Z, *erm*(A) °, ***cfr* °°**, *fex*A °°, *tet*(K), *grlA* (p.S80F), *gyrA* (p.S84L)
76669/3 ^#^	2010	Nasal swab	Pig finishing	t034	398	398	Vc	CHL, CIP, TET, TRI, FOX, PEN, TML	*mec*A, *bla*Z, *vga*(A)V, *cfr* *°°, *fex*A °°, *tet*(K), *tet*(M), *dfr*G, *grlA* (p.S80F), *gyrA* (p.S84L)
55864/23 ^§^	2011	Dust sample	Pig breeding and finishing	t127	1	1	V	CHL, CIP, GEN, KAN, TET, FOX, CLI, ERY, PEN	*aac*(6′)-*aph*(2″), *mec*A, *bla*Z, *erm*(A) °, *erm*(C), *cfr* *°°, *fex*A °°, *tet*(K), *grlA* (p.S80F), *gyrA* (p.S84L)

ST: sequence type; CC: clonal complex; CHL: chloramphenicol; CIP: ciprofloxacin; GEN: gentamicin; KAN: kanamycin; TET: tetracycline; FOX: cefoxitin; CLI: clindamycin; ERY: erythromycin; PEN: penicillin; TRI: trimethoprim; TML: tiamulin; LIZ: linezolid; SYN: synercid (quinopristin/dalfopristin). ^§^ Alba et al., 2015. [16] ^#^ Feltrin et al., 2016. [17] * Identity < 100% with the *cfr* reference sequence (AM408573) of CGE ResFinder database. ° Located on the same contig. °° Located on the same contig.

## Data Availability

The whole genome sequence data presented in this study were deposited in the European Nucleotide Archive (http://www.ebi.ac.uk/ena (accessed on 27 February 2023)) under the study accession number PRJEB59270 with the following individual accession numbers: ERS14687029 (pMOL21554); ERS14687032 (55864/23); ERS14687030 (21554/1); and ERS14687031 (76669/3).

## Supplementary Materials

The following supporting information can be downloaded at: https://www.mdpi.com/article/10.3390/antibiotics12030530/s1, Table S1: Bakta annotations; Table S2: MobileElementFinder results.
